# Longitudinal Point Prevalence Survey of Antimicrobial Consumption in Russian Hospitals: Results of the Global-PPS Project

**DOI:** 10.3390/antibiotics9080446

**Published:** 2020-07-25

**Authors:** Svetlana Rachina, Yuliya Belkova, Roman Kozlov, Ann Versporten, Ines Pauwels, Herman Goossens, Elena Bochanova, Olga Domanskaya, Elena Elokhina, Ludmila Ezhova, Vladimir Mishchenko, Oksana Ni, Dmitry Popov, Ulyana Portnjagina, Evgeny Shchetinin, Vera Shegimova, Yulia Strezh, Vera Vityazeva, Nadezhda Zubareva, Russian Global-PPS Project Study Group

**Affiliations:** 1 Internal Medicine Department, First Moscow State Medical University, 119991 Moscow, Russia; 2 Institute of Antimicrobial Chemotherapy, Smolensk State Medical University, 214019 Smolensk, Russia; Yuliya.Belkova@antibiotic.ru (Y.B.); Roman.Kozlov@antibiotic.ru (R.K.); 3 Vaccine & Infectious Disease Institute, University of Antwerp, 2610 Antwerp, Belgium; ann.versporten@uantwerpen.be (A.V.); Ines.Pauwels@uantwerpen.be (I.P.); Herman.Goossens@uza.be (H.G.); 4 Department of Pharmacology and Pharmaceutical Consulting, Krasnoyarsk State Medical University n.a. Professor V. F. Voyno-Yasenetsky, 660022 Krasnoyarsk, Russia; bochanova@list.ru; 5 Kuzbas Children’s Clinical Hospital n.a. Professor Y. Е. Malachovskiy, 654063 Novokuznetsk, Russia; olga-domanskaya@mail.ru; 6 Omsk Regional Clinical Hospital, 644111 Omsk, Russia; elochina@yandex.ru; 7 City Clinical Hospital n.a. V. V. Vinogradov, 117292 Moscow, Russia; ezhovalg@mail.ru; 8 Federal Centre of Traumatology, Orthopedics and Endoprosthesis Replacement, 214019 Smolensk, Russia; vladimir.m.mischenko@gmail.com; 9 Regional Clinical Hospital no.2, 350012 Krasnodar, Russia; ni.oksana@gmail.com; 10 National Medical Research Center for Cardiovascular Surgery n.a. A.N. Bakulev, 121552 Moscow, Russia; da_popov@inbox.ru; 11 Department of Internal Medicine and General Medical Practice (Family Medicine), North-Eastern Federal University, 677007 Yakutsk, Russia; Ulyana-nsk@mail.ru; 12 Department of Pathological Physiology, Stavropol State Medical University, 355017 Stavropol, Russia; ev.cliph@rambler.ru; 13 Republican Clinical Hospital n.a. N.A. Semashko, 670031 Ulan-Ude, Russia; shegimova@mail.ru; 14 Tomsk Regional Clinical Hospital, 634063 Tomsk, Russia; j_strezh@mail.ru; 15 Republican Children’s Hospital, 185000 Petrozavodsk, Russia; vityazeva5@gmail.com; 16 General Surgery Department #1, Perm State Medical University n.a. E.A. Vagner, 614000 Perm, Russia; nzubareva07@mail.ru

**Keywords:** antimicrobials, pharmacoepidemiology, point prevalence survey, inpatients

## Abstract

Antimicrobial resistance is one of the key issues limiting the successful treatment of infectious diseases and associated with adverse medical, social and economic consequences on a global scale. The present study aims to evaluate antimicrobials prescribing patterns and assess progress in quality indicators in Russian multidisciplinary hospitals using three repetitive point prevalence studies (PPSs) over 4 years (Global-PPS 2015, 2017 and 2018). Out of 13,595 patients from 21 hospitals surveyed over the three time points, 3542 (26.14%) received antimicrobials, predominantly third-generation cephalosporins (44.7% in 2015, 34.1% in 2017 and 41.8% in 2018). Compliance with the hospital antibiotic guidelines was 74.8%, 66.8% and 74.3%, respectively. Indication for treatment was recorded in 72.6%, 84.1% and 82.6%, while stop/review date was documented only in 40.5%, 46.5% and 61.1% of cases. Perioperative antibiotic prophylaxis exceeded 1 day in 92%, 84% and 81% of cases. Targeted therapy rate at all time points did not exceed 15.1%, treatment based on the biomarkers rate—19.9%. For the part of PPS-2017 and 2018 analyzed in dynamics, no prominent trends were noted. The results of the project provide the basis for the development of appropriate antimicrobial stewardship programs tailored according to local practices for each hospital in the project.

## 1. Introduction

Antimicrobial resistance is one of the key issues limiting the successful treatment of infectious diseases and associated with adverse medical, social and economic consequences on a global scale [1,2]. In recent decades a significant increase in the prevalence of multiresistant microorganisms, primarily Enterobacterales producing extended-spectrum beta-lactamases (ESBLs), carbapenem-resistant strains of *Pseudomonas aeruginosa* and *Acinetobacter baumanii* and methicillin-resistant strains of *Staphylococcus aureus* has been observed in Russian hospitals [3,4,5,6].

According to a multicenter study [3], covering 49 hospitals in 26 cities of the Russian Federation, the percentage of ESBL-producing nosocomial strains of entrobacteria in 2015-2016 exceeded 67% in addition to high levels of resistance to non-beta-lactam such as aminoglycosides (up to 61.1%) and fluoroquinolones (69.5%). Even though carbapenems remain active against the majority of nosocomial enterobacteria strains (89.5%–90%), the increase in the proportion of isolates resistant to drugs of this group due to the production of carbapenemases (14.4%) is alarming. An increase in resistance to carbapenems was also noted among nosocomial isolates of *P. aeruginosa* (67.5% for imipenem and 55.5% for meropenem) [4] and *A. baumannii* (77.4% and 77.1% respectively) [5].

One of the leading causes for the increase in resistance to antimicrobial drugs (AMDs) in medical institutions is irrational use, which accounts for up to 50% of all AMD prescriptions [7]. Point prevalence studies (PPSs) have established themselves as a convenient, low-cost, and at the same time standardized and validated tool for monitoring the prescribing of drugs in inpatients [8].

The Global-PPS project was planned as a universal tool that allows us to obtain information on the use of AMD in hospitalized patients, to reveal the main problems and develop targeted measures as part of local antimicrobial stewardship programs and monitor the effectiveness of their implementation [9,10]. The project started in 2014 with the data from 335 health facilities in 53 countries of the world and escalated up to 735 hospitals from 75 countries by 2018 [11]. As recently as 2015, the Russian Federation joined the Project [12]. This article presents detailed Russian results of the Global-PPS project for the period from 2015 till 2018.

The present study aims to evaluate antimicrobials prescribing patterns and assess the progress in quality indicators of antimicrobial prescribing in Russian multidisciplinary hospitals using three repetitive PPSs over 4 years (Global-PPS 2015, 2017 and 2018).

## 2. Results

### 2.1. Characteristics of the Hospitals and Study Population

The main characteristics of the health facilities and the patient population included in each of the PPS are presented in Table 1.

Fifteen different hospitals participated to the PPS of which 33% at least twice. Only one hospital took part in all three surveys, one in the first and third survey and three in the second and third survey. In each of the time spans, seven hospitals were included. About half of the hospitals in each PPS turned out to be secondary ones. The total patient population in the projects reached up to 13,595. Population characteristics in different PPS were relatively uniform except for the age. Thus in the study carried out in 2015, the percentage of adult patients was 63.9%, while in subsequent years this figure reached 95% or more.

### 2.2. General Trends of Systemic AMD Prescribing

The average share of patients in Russian hospitals receiving AMDs on the day of PPS was 26.1% with variations less than 5% between different years and in different age groups. The only exclusion was the proportion of newborns receiving AMDs in 2017 (51.2%), which was due to the peculiarities of hospitals included in the project in that year.

Despite the significant variability of the results, it was possible to identify general trends in the prevalence of AMD prescribing depending on the by type of a ward (Table 2). Thus in medical wards this indicator was the lowest and did not exceed 20% (except for neonatal ones in 2017), in surgical wards it was higher: 23.9%–38.1%, while in intensive care units (ICU) the proportion of patients receiving AMDs was the highest (56.6%–100%).

**Figure 1:**
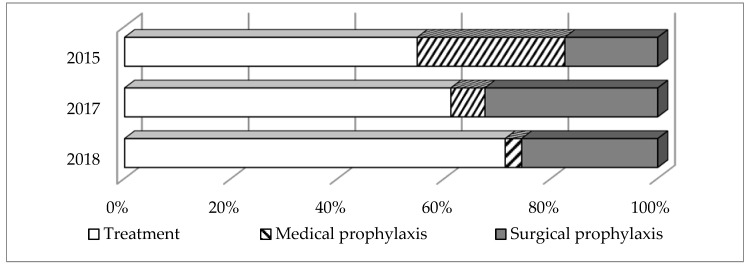
Antimicrobials use by indication.

The main indications for systemic AMD are presented in Figure 1, the 10 most common diagnoses treated with therapeutic antimicrobials, in Table 3. The therapeutic use of systemic AMD prevailed in all PPS (54.9% in 2015, 61.2% in 2017, and 71.4% in 2018, which corresponded to 13.9%, 14.1% and 21.5% of the total number of hospitalized patients, respectively). The prevalence of perioperative antibiotic prophylaxis was 17.4%, 32.4%, and 25.5% respectively (4.4%, 7.5% and 7.7% of hospital admissions), while the prevalence of medical prophylaxis was much higher in 2015 (27.7%, which accounted for 7% of hospitalized patients), and did not exceed 6.4% in 2017 and 3.1% in 2018 (1.5% and 0.9% of hospital admissions, respectively).

Most antimicrobials for therapeutic use were prescribed to treat pneumonia/lower respiratory tract infection, skin and soft tissue infections and upper urinary tract infections (Table 3).

Most systemic antibacterials (ATC J01) administered in Russian hospitals included beta-lactams (73.2%, 65.4% and 55.1%, respectively), predominantly 3rd generation cephalosporins (44.7%, 34.1% and 41.8% of all AMD) and penicillins (9.8%, 15% and 11% of all AMD), followed by quinolones (10.2%, 15.8% and 16.5%) and the so-called “other antibacterials” (7.5%, 9.9% and 8.7%), mainly metronidazole, vancomycin and nitrofurans (Table 4). Significant differences in the frequency of use were noted only for 2nd generation cephalosporins, the proportion of which decreased from 7.2% in 2015 to 0.4% in 2017 and 0.1% in 2018.

### 2.3. Key Patterns and Quality Indicators of Systemic AMD Prescribing

Analysis of key patterns and quality indicators of systemic AMD prescribing (Table 5) confirmed the predominance of intravenous therapy in Russian hospitals (85% in 2015, 84.6% in 2017, and 86.7% in 2018). The prevalence of combined therapy was 9.8%, 16.9%, and 17.9%, respectively. In the vast majority of cases (85.5%, 87.9%, and 84.9%), antimicrobials were administered empirically, and were rarely based on the level of biomarkers (19.9%, 12.1%, and 17.8%, respectively).

In most cases, drug selection complied with the hospital antibiotic guidelines (74.8%, 66.8%, and 74.3%), while the prevalence of non-compliance as well as lack of recording the indication and stop/review dates for the treatment in medical records remained too high.

**Figure 2:**
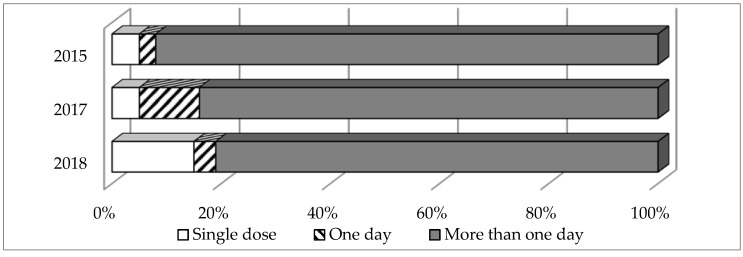
Duration of surgical prophylaxis.

Prolonged perioperative antibiotic prophylaxis was high (more than 1 day in 92% of prescriptions in 2015, 84% in 2017 and 81% in 2018). The most frequently prescribed AMD for surgical prophylaxis was a 3rd generation cephalosporin (in 28.2%, 33.4% and in 48.3% of prescriptions, respectively) (Figure 2).

### 2.4. Quality Indicators Dynamic at the Level of Study Centres

Since the hospitals included in the PPS in 2015, 2017 and 2018 were different, the analysis of a set of quality indicators in individual health facilities was performed only for 4 sites which participated in the project in 2017 and 2018 (Table 6).

Prominent variations in the value of each of the indicators between individual health facilities were noted. The dynamic at a single hospital level was relatively low although commonly positive, and usually did not exceed 15%. Compliance with hospital antibiotic guidelines improved for all hospital settings. Two hospital sites were able to document the reason for therapeutic prescription and stop/review date more often. Biomarker data were used more often to support AMD prescribing decision in three hospitals. 

## 3. Materials and Methods

Data collection for the project was performed during three consecutive PPSs, carried out from February to April 2015, from September to November 2017 and from September to November 2018 in multidisciplinary hospitals in various regions of the Russian Federation. The recruitment in the project was performed on a voluntary basis. A variety of hospitals were invited to participate but not all of them accepted.

The study was carried out according to the methodology of the Global-PPS project [12] and covered all departments in each hospital. Each ward included in the survey had to be surveyed only once in a single day and the data collection period for the hospital did not exceed 4 weeks. All the inpatients admitted to a ward at 8 a.m. on the day of the project were included in the study. The investigators registered real practice of AMD administrations performed by the staff of the hospital and had no influence on the process. The prevalence of antimicrobials prescription was calculated by dividing the number of patients treated with AMD by the total number of inpatients surveyed.

Drugs were classified according to the standardized WHO Anatomical Therapeutic Chemical (ATC) classification system [13]. Detailed data on the antimicrobial agent, age and gender, indication for treatment were collected for each patient receiving at least one antimicrobial for a prophylactic or therapeutic purpose. Antimicrobials included: antibacterials for systemic use (ATC J01), antimycotics and antifungals for systemic use (J02 and D01BA), drugs for the treatment of tuberculosis (J04A), antibiotics used as intestinal anti-infectives (A07AA), antiprotozoals used as antibacterial agents, nitroimidazole derivatives (P01AB), antivirals for systemic use (J05) and antimalarials (P01B).

The prescription of AMD in clinical practice was evaluated by means of quality indicators specified by the Global-PPS international study protocol:

1. duration of perioperative prophylaxis,
2. compliance with local hospital guidelines (customized protocols of AMD prescribing based on the Russian National guidelines [14,15,16,17,18,19,20]),
3. documentation of indication for prescription of antibiotic therapy,
4. documentation of stop/review date,
5. targeted treatment based upon microbiological result,
6. treatment based upon the use of biomarker data (C-reactive protein, procalcitonin, or other).

Full information on the method used is available on the website: www.global-pps.com.

The data were entered by the participating hospitals in the web-based application of the Global-PPS with the database hosted at the University of Antwerp, Belgium. The data were analyzed by means of descriptive statistics.

## 4. Discussion

For this article, we present results of three consecutive PPSs undertaken in Russian multidisciplinary hospitals in 2015, 2017 and 2018. It should be noted that, although all three PPSs included in the publication were carried out by a uniform methodology, our possibility to carry out a comparative assessment of the results over the years was limited as most hospitals participated only once.

Out of 13,595 patients from 21 hospitals surveyed over the three time points, 26.14% received antimicrobials, predominantly third-generation cephalosporins (44.7% in 2015, 34.1% in 2017 and 41.8% in 2018). Compliance with the hospital antibiotic guidelines was 74.8%, 66.8% and 74.3%, respectively. Indication for treatment was recorded in 72.6%, 84.1% and 82.6%, while stop/review date was documented only in 40.5%, 46.5% and 61.1% of cases. Perioperative antibiotic prophylaxis exceeded 1 day in 92%, 84% and 81% of cases.

The results of all PPSs demonstrate moderate levels of systemic AMD usage in Russian hospitals regardless of the age of patients. As expected, the highest consumption levels were observed in ICU. At the same time, the levels of AMD usage were lower than in other regions of the world (25.4% in the Russian Federation vs. 28.1% in the countries of Western Europe vs. 34.4% in the countries of Northern Europe vs. 38.6% in the countries of North America in 2015 [12], 23.1% in the Russian Federation in 2017 vs. 30.5% in European Union/European Economic Area in 2016–2017 [21]).

In spite of the fact that some variations in the proportion of patients who received AMD (25.4% in 2015 vs. 23.1% in 2017 vs. 30.1% in 2018) were revealed, different lists of hospitals that participated in the project do not allow us to draw a conclusion whether the changes are a consequence of the increasing frequency of drugs use or are accidental and related to the peculiarities of the hospitals included in each of the PPS. Monitoring this quality indicator during subsequent PPS will allow for a more accurate assessment of the significance of these changes.

Although the majority of AMDs were prescribed for therapeutic purposes, in 2015 a relatively high frequency of drug use was noted for medical prophylaxis, which, given the small list of indications, requires more detailed investigation to assess the rationality and may become one of the potential points to correct the excessive use of AMDs. In subsequent years, the proportion of patients receiving AMDs for medical prophylaxis significantly decreased (from 27.7% in 2015 to 3.1% in 2018), while the proportion of patients receiving these drugs for therapeutic purposes and surgical prophylaxis significantly increased (from 54.9% to 71.4% and from 17.4% to 25.5% respectively). Such changes may be the result of the introduction of local programs in hospitals aimed to optimize antimicrobial therapy for certain diseases, as was shown in the study of Kopczynska, et al. [22]. However, the information available is not sufficient to clearly link this trend with any particular interventions in the hospitals involved in the project.

The high frequency of beta-lactam prescribing in Russian hospitals was confirmed in the current project (55.1%–73.2%). According to other studies with the similar methodology, beta-lactams remain the leading group of AMDs in Europe and Central Asia [23]. Noteworthy is the leading position of 3rd generation cephalosporins, whose share in all the years of PPS has remained stably high (44.7% in 2015, 34.1% in 2017 and 41.8% in 2018). One of the reasons for such prominent consumption of 3rd generation cephalosporins is the common use of ceftriaxone as a part of perioperative antimicrobial prophylaxis (in 28%–48% of cases). At the same time, the use of 3rd generation cephalosporins for this purpose is not recommended due to the lack of significant advantages in efficacy over 1st and 2nd generation cephalosporins and high potential of resistance selection [24]. This finding provides an opportunity to optimize the use of systemic AMDs as well as the reduction of the perioperative antimicrobial prophylaxis duration to the recommended single doses since the prolongation for a day or more does not lead to improvement in prevention of infectious complications [24].

According to WHO AWaRe classification of antibiotics [25] 3rd generation cephalosporins belong to the Watch group and are considered to be the key targets of stewardship programs and monitoring. The next most prevalent class of drugs—fluoroquinolones—fall into the same category. An increase in the frequency of quinolones use identified during the project (10.2% in 2015 vs. 15.8% in 2017 vs. 16.5% in 2018) is consistent with the general trend of increasing consumption of drugs of this group in Russia and can be explained by a wide range of registered indications for both treatment and prophylaxis of infections in hospitalized adults and availability of a large number of cheap generics of levofloxacin. Though the Global-PPS Project methodology does not allow us to evaluate the rationality of drugs prescription in individual patients, the practice of widespread use of AMDs of these groups needs to be reassessed due to high risks of *Clostridium difficile*-associated diarrhea and selection of resistance, especially in gram-negative microorganisms [26,27,28].

A significant advantage of the Global-PPS project is a uniform system of quality indicators that allows us to evaluate key aspects of AMD prescribing. Compliance with the quality indicators in the Russian hospitals that have participated in the Project needs improvement although it corresponds to the average levels for all regions of the world. Thus according to the results of Global-PPS-2015, targeted therapy in Russian hospitals was observed in 14.5% of cases vs. 19.8% globally; compliance with the hospital antibiotic guidelines—in 74.8% vs. 77.4%; indication was recorded in 72.6% vs. 76.9%; stop/review date was documented in 40.5% vs. 38.3%, respectively [12]. Similar results were obtained in another study based on PPS methodology, carried out in European acute care hospitals in 2016–2017 [21]. The reason for AMD prescribing was documented in the patients’ medical records for 80.2% prescriptions vs. 84.1% in Russian hospitals in 2017. However, information about change of the antimicrobial during the infection episode was reported for 76.8% of antimicrobial prescriptions vs. 46.5% in Russian hospitals in 2017.

In total, the dynamic in these indicators during the period from 2015 to 2018 was positive, but not significant enough for all indicators except for stop/review date documentation, that increased by almost 50% of the initial level (40.5% in 2015 vs. 61.1% in 2018). 

Prominent variations in the value of each of the indicators between individual health facilities were noted probably due to the differences in the hospitals’ location, local AMD prescribing policies, stages of antimicrobial stewardship programs implementation, and levels of administrative support of such programs. Some sites, e.g., site #2, showed the best results in the majority of indicators, while other sites, e.g., site #4, were better in some, like recording the indication for treatment and biomarker data usage, but had the worst results for compliance with the hospital antibiotic guidelines and stop/review date documentation. 

Despite the existence of national recommendations for optimizing the use of AMD [29], the degree of their implementation in Russian hospitals still leaves much to be desired (up to 60 hospitals from different regions of the Russian Federation at the time of publication of the latest version of the document). Many hospitals still do not have a universal policy for the use of AMD and resistance control. The national recommendations have proven to be an effective tool for combating irrational practices of AMD usage [30,31]. It can be assumed that the improvement in antibiotic policy in some hospitals is partly associated with their participation in the former Global PPS.

Increasing the frequency of targeted therapy, compliance with the local antibiotic guidelines, documenting the rationale for AMD administration and the timing of its discontinuation or drug change, remain key priorities that should be considered in antimicrobial stewardship programs for Russian hospitals.

Although Global-PPS is not the first point-prevalence study of the use of AMDs in the Russian Federation, its significance is obvious. The project segment carried out in this country revealed some common errors that need to be corrected first, such as the widespread use of 3rd generation cephalosporins for therapeutic and prophylactic purposes, unreasonably long perioperative antibiotic prophylaxis, low rate of targeted therapy frequency, insufficiently high frequency of records in the medical documentation about the purpose and timing of AMD change/withdrawal, as well as following the recommendations when choosing the drug.

It is necessary to mention the limitations inherent in the study and directly stemming from its methodology. The data obtained during the project are generalized and provide no insight on the individual patients’ level. The project does not take into account the population and characteristics of patients, the duration and outcome of therapy, the local epidemiological situation in the hospitals, bed capacity, administrative and organizational features of the institution, regional characteristics and other factors that affect the usage of AMDs. That is why we need to be cautious while interpreting the results of the study and making comparisons between both different Russian hospitals in this country and other regions of the world.

## 5. Conclusions

An irrational approach to AMD prescribing is associated with noticeable negative medical, social and economic consequences. The Russian participation in the Global-PPS study in 2015, 2017 and 2018 presented in the current publication, revealed the most common anomalies in AMD usage in hospitalized patients such as inappropriately long-term perioperative antibiotic prophylaxis, unreasonably frequent administration of 3rd generation cephalosporin prescribed for prophylactic purposes, low rate of targeted therapy, insufficient compliance to local hospital guidelines and documenting of AMD change/stop date. The results of the project can be used to improve AMD prescribing in each of the participating hospitals as well as to monitor the effectiveness of antimicrobial stewardship interventions.

## Figures and Tables

**Table 1:** Characteristics of the hospitals included in the Global-PPS 2015, 2017 and 2018.

Characteristics	2015	2017	2018	Total
Number of hospitals, n	7	7	7	21
• primary hospitals	0	0	2	2
• secondary hospitals	3	5	4	12
• tertiary hospitals	1	1	1	3
• specialized hospitals	0	1	0	1
• paediatric hospitals	3	0	0	3
Number of beds, n	3976	6359	5610	15,945
Number of patients, n	3546	5438	4611	13,595
• adult %	63.9	95.0	96.9	87.5
• pediatric %	33.6	3.5	3.1	11.2
• neonatal %	2.6	1.5	0.0	1.3
Number of treated patients, n	899	1255	1388	3542
Number of treated patients %	25.4	23.1	30.1	26.1
• adult, %	26.6	22.5	30.3	26.2
• pediatric %	22.5	26.4	24.5	23.2
• neonatal %	31.9	51.9	0	41.3

**Table 2:** Overall antimicrobial prevalence by type of ward (%).

Type of Ward	2015	2017	2018
**Medical ward**			
adult	13.8	15.5	18.0
pediatric	15.7	6.2	1.8
neonatal	18.9	35.9	0
**Surgical ward**			
adult	30.5	25.6	38.1
pediatric	23.9	27.9	37.3
**ICU**			
adult	68.8	56.6	59.5
pediatric	97.9	40.0	100.0 ^1^
neonatal	88.2	66.7	0

^1^ a total of three patients.

**Table 3:** The 10 most common diagnoses treated with therapeutic antimicrobials (%).

Indication	2015	2017	2018
Pneumonia or lower respiratory tract infection	25.8	23.4	21.1
Skin and soft tissue infection	18.6	10.4	19.5
Upper urinary tract infection	6.6	11.2	11.4
Ear, nose and throat infection	8.5	11.2	5.9
Bronchitis	11.2	5.3	8.8
Intra-abdominal infection	-	7.6	7.7
Bone/joint infection	4.7	3.6	6.2
Gastrointestinal infection	8.3	-	-
Obstetric/gynaecological infection	-	3.2	2.5
Lower urinary tract infection	0.8	-	4
Eye infection	1.9	-	-
Sepsis or septic shock with no clear anatomic site	1.1	0.4	-
Infection of the central nervous system	-	-	1.4

**Table 4:** Overall proportional use of systemic antibacterials (ATC J01), %.

Antibacterials	2015	2017	2018
Penicillins	9.8	15.0	11.0
First-generation cephalosporins	4.6	9.6	5.8
Second-generation cephalosporins	7.2	0.4	0.1
Third-generation cephalosporins	44.7	34.1	41.8
Fourth-generation cephalosporins	2.3	0.4	3.0
Carbapenems	4.6	5.9	4.4
Quinolones	10.2	15.8	16.5
Aminoglycosides	3.7	4	3.7
Macrolides, Lincosamides, and Streptogramins	3.0	2.8	2.9
Sulfonamides and Trimethoprim	2.2	1.8	1.5
Other antibacterials	7.5	9.9	8.7

**Table 5:** Key patterns and quality indicators of systemic antimicrobial drug (AMD) prescribing, %.

Patterns	2015	2017	2018
Intravenous therapy	85.0	84.6	86.7
Multiple antimicrobials per patient	9.8	16.9	17.9
Targeted therapy ^1^	14.5	12.1	15.1
Treatment based on biomarker data	19.9	12.1	17.8
Compliance with the hospital antibiotic guidelines	74.8	66.8	74.3
Indication for treatment was recorded	72.6	84.1	82.6
Stop/review date documented	40.5	46.5	61.1

^1^ prophylactic prescribing is excluded

**Table 6:** Quality indicators of systemic AMD prescribing in four hospital sites with repetitive PPS in 2017 and 2018, %.

Quality Indicator	2017	2018
**Compliance with the hospital antibiotic guidelines**		
Site #1	52.5	60.7
Site #2	92.6	95.8
Site #3	68.7	88.6
Site #4	47.9	60.4
**Indication for treatment was recorded**		
Site #1	63.3	72.0
Site #2	97.9	97.3
Site #3	79.4	86.4
Site #4	92.9	86.8
**Stop/review date documented**		
Site #1	23.5	25.5
Site #2	98.5	97.5
Site #3	36.5	42.4
Site #4	15.4	9.9
**Treatment based on biomarker data**		
Site #1	0.5	13.8
Site #2	0.0	14.7
Site #3	0.9	0.0
Site #4	17.8	19.8
